# Genome-wide identification of *BcGRF* genes in flowering Chinese cabbage and preliminary functional analysis of BcGRF8 in nitrogen metabolism

**DOI:** 10.3389/fpls.2023.1144748

**Published:** 2023-03-09

**Authors:** Shuaiwei Zhang, Guangguang Li, Yudan Wang, Ali Anwar, Bin He, Jiewen Zhang, Changming Chen, Yanwei Hao, Riyuan Chen, Shiwei Song

**Affiliations:** ^1^ College of Horticulture, South China Agricultural University, Guangzhou, China; ^2^ Guangzhou Institute of Agriculture Science, Guangzhou, China

**Keywords:** GRF, genome-wide identification, NRT1.1, nitrate signaling, transcriptional regulation, flowering Chinese cabbage

## Abstract

Growth-regulating factors (GRFs) are a unique family of transcription factors with well-characterized functions in plant growth and development. However, few studies have evaluated their roles in the absorption and assimilation of nitrate. In this study, we characterized the *GRF* family genes of flowering Chinese cabbage (*Brassica campestris*), an important vegetable crop in South China. Using bioinformatics methods, we identified *BcGRF* genes and analyzed their evolutionary relationships, conserved motifs, and sequence characteristics. Through genome-wide analysis, we identified 17 *BcGRF* genes distributed on seven chromosomes. A phylogenetic analysis revealed that the *BcGRF* genes could be categorized into five subfamilies. RT-qPCR analysis showed that *BcGRF1*, *8*, *10*, and *17* expression clearly increased in response to nitrogen (N) deficiency, particularly at 8 h after treatment. *BcGRF8* expression was the most sensitive to N deficiency and was significantly correlated with the expression patterns of most key genes related to N metabolism. Using yeast one-hybrid and dual-luciferase assays, we discovered that BcGRF8 strongly enhances the driving activity of the *BcNRT1.1* gene promoter. Next, we investigated the molecular mechanism by which *BcGRF8* participates in nitrate assimilation and N signaling pathways by expressing it in Arabidopsis. BcGRF8 was localized in the cell nucleus and *BcGRF8* overexpression significantly increased the shoot and root fresh weights, seedling root length, and lateral root number in Arabidopsis. In addition, *BcGRF8* overexpression considerably reduced the nitrate contents under both nitrate-poor and -rich conditions in Arabidopsis. Finally, we found that BcGRF8 broadly regulates genes related to N uptake, utilization, and signaling. Our results demonstrate that BcGRF8 substantially accelerates plant growth and nitrate assimilation under both nitrate-poor and -rich conditions by increasing the number of lateral roots and the expression of genes involved in N uptake and assimilation, providing a basis for crop improvement.

## 1 Introduction

Nitrogen (N) affects plant growth and development, including leaf and root growth and development (Rahayu et al., 2005; Forde, 2014), seed germination and dormancy (Matakiadis et al., 2009), and floral induction (Castro Marín et al., 2011). N directly affects photosynthesis by affecting the chlorophyll content and photorespiration (Konnerup and Brix, 2010). It influences root water uptake by regulating stomatal conductance and abscisic acid contents and regulates stress resistance by modulating the mineral nutrient absorption capacity (Zhang and Chen, 2014). In addition, N acts as a signaling molecule affecting plant physiological, biochemical, and morphological processes, including gene-specific expression, redox status, root morphology, and ion uptake (Bloom, 2015; Li, L. et al., 2021). N metabolism encompasses the entire process of N absorption, assimilation, and utilization in plants (Zhang et al., 2021). Nitrate ( ${\text{NO}}_{3}^{-}$ ) is a major source of N for terrestrial plants and rapidly induces changes in gene expression, N metabolism, and accumulation (Gojon et al., 2009). After ${\text{NO}}_{3}^{-}$ enters plant cells through ${\text{NO}}_{3}^{-}$ transporters (NRTs), it is first reduced to nitrite ions ( ${\text{NO}}_{2}^{-}$ ) by ${\text{NO}}_{3}^{-}$ reductase (NR). ${\text{NO}}_{2}^{-}$ translocates to the plastids, where it is reduced to ammonium ( ${\text{NH}}_{4}^{+}$ ), the final form of inorganic N, by ${\text{NO}}_{2}^{-}$ reductase (NIR) (Li et al., 2017). Various enzymes and transporters are involved in N metabolism; therefore, altering the expression of a single gene involved in N uptake or assimilation may not significantly improve N use efficiency (NUE) (Ueda and Yanagisawa, 2018). To coordinately modify the expression of multiple genes related to a desired trait, modifying the expression of transcription factors (TFs) is an effective approach. TFs play important roles in N uptake, transport, and assimilation; therefore, it is crucial to investigate their biological functions and regulatory role in N metabolism (Maurya et al., 2020). Advances in plant science and molecular biology techniques have facilitated the identification of various TFs with the potential to improve NUE, including v-myb avian myeloblastosis viral oncogene homolog (MYB), MADS-box, lateral organ boundary domain (LBD), NIN-like protein (NLP), basic leucine zipper protein (bZIP), NAC, DNA binding with one finger (DOF), and growth-regulating factor (GRF).

GRFs are a unique family of TFs involved in plant growth, development, and stress responses (Lee et al., 2015). Since the discovery of *OsGRF1* in rice (*Oryza sativa*) in 2000 (Knaap et al., 2000), additional GRF family genes have been identified in various plant species, including *Arabidopsis thaliana* (Kim et al., 2003), *Brassica rapa* (Wang et al., 2014), *Brassica napus* (Ma et al., 2017), *Cucumis sativus* (Zhou et al., 2018), *Solanum lycopersicum* (Khatun et al., 2017), *Nicotiana tabacum* (Zhang et al., 2018), *O. sativa* (Choi et al., 2004), *Zea mays* (Wu et al., 2014), *Triticum aestivum* Linn. (Huang et al., 2021), and *Arachis hypogaea* (Zhao et al., 2019). *GRF* genes are expressed in fast-growing and developing tissues (e.g., stem tips, flower buds, and young leaves), with weak expression in mature tissues and organs (Lee et al., 2015). GRFs play important regulatory roles in the development of roots, stems, leaves, floral organs, and seeds (Bazin et al., 2013; Suzanne et al., 2014; Wu et al., 2014). For example, the silencing of *OsGRF3*, *OsGRF4*, and *OsGRF5* in rice resulted in plants having a dwarf phenotype and delayed inflorescence formation (Suzanne et al., 2014). Some GRFs improve the NUE of crops. For example, OsGRF4 and the growth inhibitor DELLA synergistically regulate plant growth by regulating carbon and N metabolic pathways in rice (Li et al., 2018b). OsGRF4 and GRF-interacting factor 1 (OsGIF1) interact to activate the transcription of N uptake-related genes (Ammonium transporter; *OsAMT1.1* and *OsAMT1.2*) and N assimilation-related genes (*GS1*) (Ma and Liu, 2018). In addition, OsGRF4 can regulate the expression of *MYB61* to directly affect cellulose biosynthesis, and its expression is induced by low-N stress (Gao et al., 2020). Thus, the OsGRF4-MYB61 signaling pathway is associated with N metabolism. In rice, *miR396e* and *miR396f* mutants showed improved tolerance to low-N stress and exhibited high and stable yield characteristics under low-N conditions, indicating that *miR396*, an upstream regulator of *OsGRF4*, *OsGRF6*, and *OsGRF8*, indirectly regulates N absorption and assimilation (Zhang et al., 2020). In poplar, PpnGRF5-1 may regulate Nitrate transporter (*NRT*) genes *via* the TF PagLBD38 (Wu et al., 2021). However, the precise effects of GRFs on crop NUE and their mechanisms of action remain unclear.

Flowering Chinese cabbage (*Brassica campestris* L. ssp. *chinensis* var. *utilis* Tsen et Lee), a subspecies of *B. rapa* in the family Brassicaceae or Cruciferae, is an important vegetable in South China. Chinese cabbage, grown for its leafy head, shows distinct growth and development characteristics from those of flowering Chinese cabbage. The product organs/tissues of flowering Chinese cabbage are its leaves and stalks, the development of which is influenced by various factors, including temperature, hormones, and N (Song et al., 2019; Kou et al., 2021; Wang et al., 2021; Zhu et al., 2021). Flowering Chinese cabbage requires substantial N fertilization and its product organs readily accumulate ${\text{NO}}_{3}^{-}$ during production (Song et al., 2017). At present, promoting the growth of flowering Chinese cabbage, improving product quality, and reducing the amount of N fertilizer by increasing NUE remain technical challenges in flowering Chinese cabbage production because the molecular mechanism by which TFs regulate N metabolism in horticultural plants is not well understood and most research has focused on key functional genes in N metabolic pathways. Therefore, research on the regulatory mechanisms of N absorption and assimilation will help the development of technologies to improve N utilization in flowering Chinese cabbage, which has important economic value and application prospects, as well as provide theoretical support for the study of N metabolism in other leafy vegetables. In this study, we analyzed the expression patterns of *BcGRF* family genes in flowering Chinese cabbage under various N treatments and explored the regulatory pathways of key BcGRF members and their biological functions in Arabidopsis.

## 2 Materials and methods

### 2.1 Identification of *BcGRF* genes

The genome sequence and annotation files for *B. campestris* were obtained from an unpublished genome database assembled at the Department of Vegetables, College of Horticulture, South China Agricultural University (Guangdong, China). The Arabidopsis AtGRF protein sequences were downloaded from the Plant Transcription Factor Database as references (http://planttfdb.gao-lab.org/) (**Supplementary Table S1**) (Jin et al., 2017). The *B. campestris* genome was searched for *GRF* orthologs using the Blast GUI Wrapper function in TBtools, with default parameters, except for the tolerance, which was set to 0.001 (Chen et al., 2020). Possible conserved domains in candidate GRFs were identified using the CD-Search Tool (https://www.ncbi.nlm.nih.gov/Structure/bwrpsb/bwrpsb.cgi) (Ma et al., 2015), and incorrectly predicted genes and redundant sequences were manually removed. The *BcGRF* genes were named following a previously proposed nomenclature scheme (Kim et al., 2012). The sequence length, molecular weight (MW), and isoelectric point (pI) of putative BcGRFs were predicted using the ExPASy website (http://web.expasy.org/protparam/) (Li et al., 2018a).

### 2.2 Bioinformatics analysis of *BcGRF* genes

Multiple amino acid (aa) sequence alignments of BcGRF proteins were constructed using DNAMAN (DNAMAN v6.0, Lynnon Biosoft, USA). Gene structures were analyzed using the Gene Structure Display Server (GSDS) (http://gsds.gao-lab.org/index.php). An unrooted neighbor-joining phylogenetic tree was constructed using ClustalW in MEGA 7 with default parameters; bootstrap repeats were set to 1,000 (Kumar et al., 2016). Conserved motifs were predicted using the MEME program (http://meme-suite.org/tools/meme) (number of repetitions: arbitrary; maximum number of motifs: 10; and optimal width of each motif: between 6 and 100 bases). The *BcGRF* genes were mapped to chromosomes using Circos based on the physical location information in the *B. campestris* genome database (Krzywinski et al., 2009). Gene duplication events were analyzed using the multiple collinearity scan toolkit MCScanX with default parameters (Wang et al., 2012). A synteny plot of *B. campestris* and *A. thaliana* genomes and *GRF* genes was constructed using TBtools (Chen et al., 2020).

### 2.3 Expression correlation analysis

Expression correlations between *BcGRF* genes and N-related genes with significantly altered expression after N treatment (0, 2, 4, 8, 12, and 24 h) were analyzed by Pearson correlation tests and *t*-tests using the ‘PEARSON’ and ‘T.DIST’ functions in Excel (Microsoft, Seattle, WA, USA). The expression data used for this analysis were obtained from RT-qPCR analysis.

### 2.4 Yeast one-hybrid and dual-luciferase reporter assays

Yeast one-hybrid assays were conducted using the Matchmaker One-Hybrid System (630491; Clontech, Mountain View, CA, USA). The promoter of *BcNRT1.1* was divided into two parts (*proBcNTR1.1-1* and *proBcNTR1.1-2*), which were synthesized with *Kpn* I and *Sal* I flanking restriction sites and cloned into *Kpn* I- and *Sal* I-digested pAbAi bait vector (Clontech). For expression in yeast, *BcGRF8* cDNA was cloned into the pGADT7 prey vector (Clontech). *Saccharomyces* Y1H Gold (Clontech) was used as the host. Positive transformants were selected by growth on SD/-Leu medium (Clontech) containing aureobasidin A (AbA, 60231ES03; Yeasen, Shanghai, China). To analyze the transcriptional regulation of *BcNRT1.1* by BcGRF8 in *Nicotiana benthamiana* leaves, the coding sequence (CDS) of *BcGRF8* was inserted into the pGreenII 62-SK vector to generate the effector construct. To generate the reporter constructs, the two *BcNRT1.1* promoter parts were inserted into the pGreenII 0800 vector (Zhang et al., 2022). The constructs were introduced into tobacco plants *via Agrobacterium*-mediated transformation. Firefly luciferase (LUC) and *Renilla* luciferase (REN) activities were measured using a dual-luciferase reporter assay kit (E1910; Promega, Madison, WI, USA) and an Infinite M200 Pro Microplate Reader (Tecan, Männedorf, Switzerland). The LUC/REN ratio, which represents the relative activity of the promoter, was calculated. The experiment was performed three times, and each sample was tested three times. The primers are listed in **Supplementary Table S2**.

### 2.5 Subcellular localization analysis and *A. thaliana* transformation

The full-length *BcGRF8* CDS without a stop codon was subcloned into the pBI121-GFP vector and fused with green fluorescent protein (GFP) (primers are listed in **Supplementary Table S2**) (Zhang et al., 2022). pBI121-BcGRF8-GFP and pBI121-GFP control vectors were transferred into *Agrobacterium tumefaciens* strain GV3101 cells, which were injected into *N. benthamiana* leaves. Twenty-four hours later, the GFP signal was captured using a fluorescence microscope (Axio Imager D2; Zeiss, Jena, Germany). Transgenic Arabidopsis Col-0 plants expressing pBI121-BcGRF8-GFP were generated using the floral dip method. Positive transformants were screened on 1/2MS medium containing 50 mg/L kanamycin and identified by PCR. Homozygous transformants in the T3 generation were used for analyses.

### 2.6 Plant material and treatments

Plant experiments were carried out in a greenhouse at the Southern Facility Vegetable Experiment Base of the College of Horticulture, South China Agricultural University. Flowering Chinese cabbage seeds (cultivar ‘Youlv 501’) were provided by the Guangzhou Academy of Agricultural Science. Seedlings were grown in a sponge block, and seedlings with two true leaves and the same growth status were transferred to 18-L hydroponic containers. Each pot contained 12 randomized plants. Detailed nutrient solution information is presented in **Supplementary Table S3A**. Five organs (roots, stems, leaves, flowers, and seeds) were collected, as available, in different developmental stages (cotyledon stage, four-leaf stage, six-leaf stage, flowering stage, and fruit-setting stage). For N-deficiency treatment, NaNO_3_ in the nutrient solution was replaced with NaCl at the four-leaf stage. After seedlings were placed in an N-deficient nutrient solution for 0, 1, 2, 4, 8, 12, 24, or 48 h, leaf samples were collected. The seedlings of the same batch were then returned to the normal nutrient solution, and leaf samples were collected at 0, 2, 4, 8, 12, and 24 h. All treatments were set up with three biological replicates, and the samples were stored at –80°C.

Sterilized T3 and wild-type (WT) Arabidopsis seeds were stratified at 4°C for 2 days and plated on a modified MS solid medium. Limited-N medium was prepared by modifying the MS medium to contain KNO_3_ as the sole N source (1, 3, or 10 mM KNO_3_) (Yu et al., 2016). To observe growth rates and phenotypes under different N conditions, seedlings were grown on a medium containing different concentrations of ${\text{NO}}_{3}^{-}$ at 23–25°C under 16 h of light (100 µmol m^–2^ s^–1^) and 8 h of darkness.

For hydroponic culture, sterilized Arabidopsis (T3 and WT) seeds were germinated on 5% (*w*/*v*) agar in 1.5-mL centrifuge tube caps to fix the seedling. When the roots reached >1.2 cm, the centrifuge tube caps were placed on a support made of a thick polystyrene foam board with holes, allowing the roots to grow into a hydroponic solution containing KNO_3_ as the sole N source (0 mM KNO_3_ for N-deficient nutrient solution or 3 mM KNO_3_ for conventional nutrient solution). Detailed information on the nutrient solution is presented in **Supplementary Table S3B**. Plants were cultured at 22°C under a 16-h light/8-h dark photoperiod.

### 2.7 RNA isolation and quantitative reverse transcription (RT-q)PCR analysis

Total RNA was isolated using the Plant Total RNA Kit (LS1040; Promega (Beijing) Biotechnology Co., Ltd., Beijing, China). Complementary DNA (cDNA) was synthesized using the *Evo M-MLV* RT Kit from Ecorui (Hunan) Biotechnology Co., Ltd. (Hunan, China). qPCRs were run in a LightCycler 480 Real-Time PCR System (Roche, Basel, Switzerland) using the SYBR Green Premix *Pro Taq* HS qPCR Kit (AG11701; Accurate, Hunan, China) per the manufacturer’s instructions. Reaction mixtures contained 10 μL of 2× SYBR Green Taq, 1.0 μL of upstream and downstream primers (10 μmol/L) (**Supplementary Table S2**), 1.0 μL of cDNA template, and RNase-free H_2_O to 20.0 μL. The amplification program was 95°C for 5 min followed by 40 cycles of 95°C for 5 s and 60°C for 30 s. *GADPH* was used as an internal reference gene for normalization. Relative target gene expression levels were calculated using the 2^–ΔΔCt^ method (Livak and Schmittgen, 2001). Gene expression profiles were visualized as a heatmap using TBtools (Chen et al., 2020).

### 2.8 Determination of morpho-physiological indicators

Primary root length was measured using ImageJ. Plant fresh weight (FW) was measured using a ten-thousandth electronic analytical balance. The ${\text{NH}}_{4}^{+}$ N content was measured using the indophenol blue colorimetric method; in brief, ${\text{NH}}_{4}^{+}$ N reacts with hypochlorite and phenol in a strong alkaline medium to generate the water-soluble and color-stable dye, indophenol blue. Absorbance was measured at 625 nm (Tzollas et al., 2010). The ${\text{NO}}_{3}^{-}$ content was measured using the salicylic acid method (Vendrell and Zupancic, 1990).

## 3 Results

### 3.1 Identification of BcGRFs in flowering Chinese cabbage

**Figure 1:**
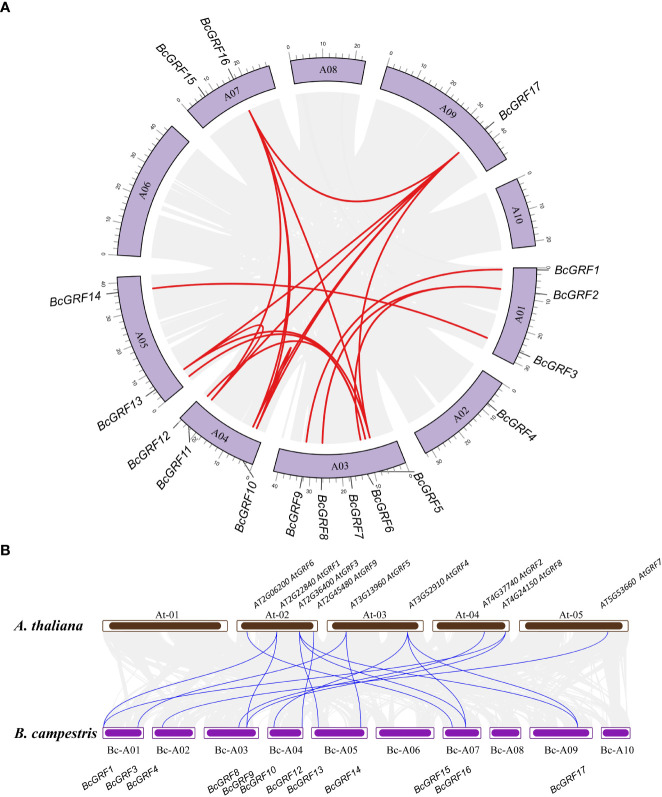
Synteny analysis of the *BcGRF* genes. **(A)** The schematic representations of the chromosomal distribution and inter-chromosomal relationships of *B*. *campestris GRF* genes. The gray lines indicate all synteny blocks in the *B*. *campestris* genome, whereas the red lines indicate duplicated *GRF* gene pairs. **(B)** Synteny analysis *B*. *campestris* and *A. thaliana GRF* genes. The gray lines in the background indicate the collinear blocks within the *B*. *campestris* and *A*. *thaliana* genomes, whereas the red lines indicate the syntenic *GRF* gene pairs.

A total of 17 BcGRF family members were identified using all AtGRF aa sequences as references. The *BcGRF* genes were named *BcGRF1*–*BcGRF17* according to the nomenclature scheme proposed by Schilling (**Table 1**). The 17 *BcGRF* genes were distributed over seven chromosomes, including five on chromosome 3 (*BcGRF5–BcGRF9*), three on chromosomes 4 (*BcGRF1–BcGRF3*) and 1 (*BcGRF10–BcGRF12*), two on chromosomes 5 (*BcGRF13* and *BcGRF14*) and 7 (*BcGRF15*, *BcGRF16*), and one on chromosomes 2 (*BcGRF4*) and 9 (*BcGRF17*). No *BcGRF* genes were found on chromosomes 6, 8, and 10 (**Figure 1A**).

**Table 1:** *GRF* family genes in *B. campestris* and basic gene and protein characteristics.

Gene name	Gene ID	CDS (bp)	Arabidopsis homolog	Protein		
				Length (aa)	MW (kDa)	pI
*BcGRF1*	Bra_cxA01g032630.1	1173	*AtGRF1, AtGRF2*	390	43226.55	6.09
*BcGRF2*	Bra_cxA01g007880.1	1344	*AtGRF8*	447	50870.22	7.39
*BcGRF3*	Bra_cxA01g045660.1	996	*AtGRF4*	331	37050.94	9.59
*BcGRF4*	Bra_cxA02g034810.1	1047	*AtGRF4*	348	38114.66	8.67
*BcGRF5*	Bra_cxA03g048510.1	1083	*AtGRF7*	360	39430.73	8.72
*BcGRF6*	Bra_cxA03g004750.1	870	*AtGRF3*	290	31744.38	9.49
*BcGRF7*	Bra_cxA03g014750.1	1410	*AtGRF1*	469	51290.16	6.88
*BcGRF8*	Bra_cxA03g030750.2	972	*AtGRF5*	323	36649.40	9.00
*BcGRF9*	Bra_cxA03g041560.1	1617	*AtGRF8*	538	57765.31	9.23
*BcGRF10*	Bra_cxA04g001300.1	1314	*AtGRF2*	437	49044.96	9.31
*BcGRF11*	Bra_cxA04g006920.1	1116	*AtGRF3*	371	40882.21	8.21
*BcGRF12*	Bra_cxA04g033250.1	1104	*AtGRF9*	367	41700.25	8.55
*BcGRF13*	Bra_cxA05g010200.1	1176	*AtGRF3, AtGRF4*	391	44108.19	8.22
*BcGRF14*	Bra_cxA05g036510.1	702	*AtGRF5*	233	26119.59	9.51
*BcGRF15*	Bra_cxA07g037430.1	867	*AtGRF5*	288	33009.45	9.26
*BcGRF16*	Bra_cxA07g022620.1	1095	*AtGRF6*	364	40551.93	8.86
*BcGRF17*	Bra_cxA09g022050.1	1146	*AtGRF3, AtGRF4*	381	42543.69	6.69

The basic characteristics of the *BcGRF* genes are listed in **Table 1**. The CDSs of the *BcGRF* genes ranged from 702 to 1617 bp in length; *BcGRF14* was the shortest (702 bp) and *BcGRF9* was the longest (1617 bp). The proteins were 233–538 aa in length, with MWs of 26.12–57.77 kDa and pI values ranging from 6.09 (*BcGRF1*) to 9.59 (*BcGRF3*) (**Table 1**).

### 3.2 Phylogenetic analysis and multiple sequence alignment of BcGRFs

**Figure 2:**
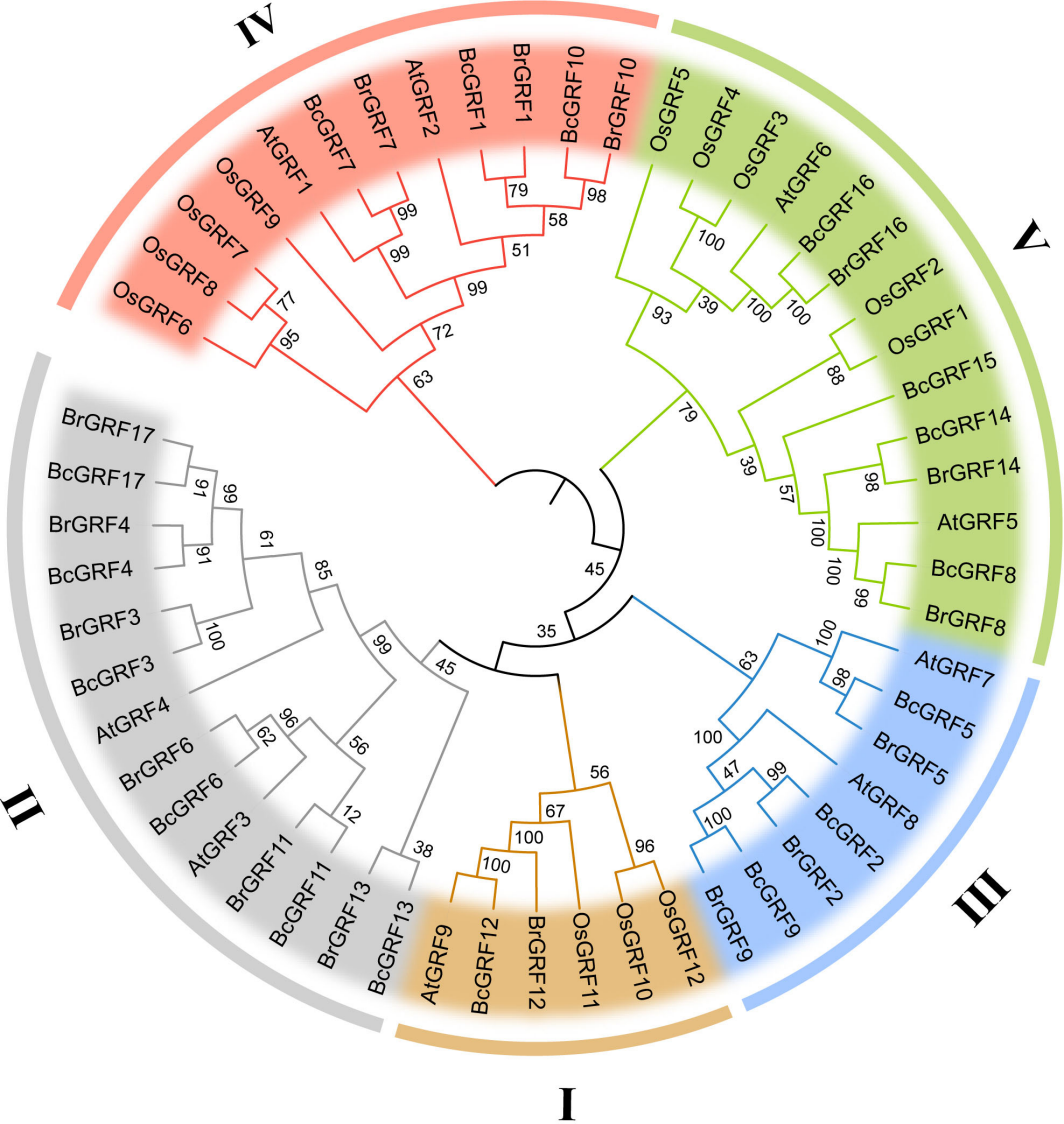
The unrooted phylogenetic tree of the *GRF* gene family in *B. campestris* (Bc), *A. thaliana* (At), *B*. *rapa* (Br), and *O. sativa* (Os). The different colors represent the five GRF subfamilies (I, II, III, IV, and V). Blue represents the GRFs from *B*. *campestris*. The values at the nodes indicate bootstrap support based on 1000 replicates.

**Figure 3:**
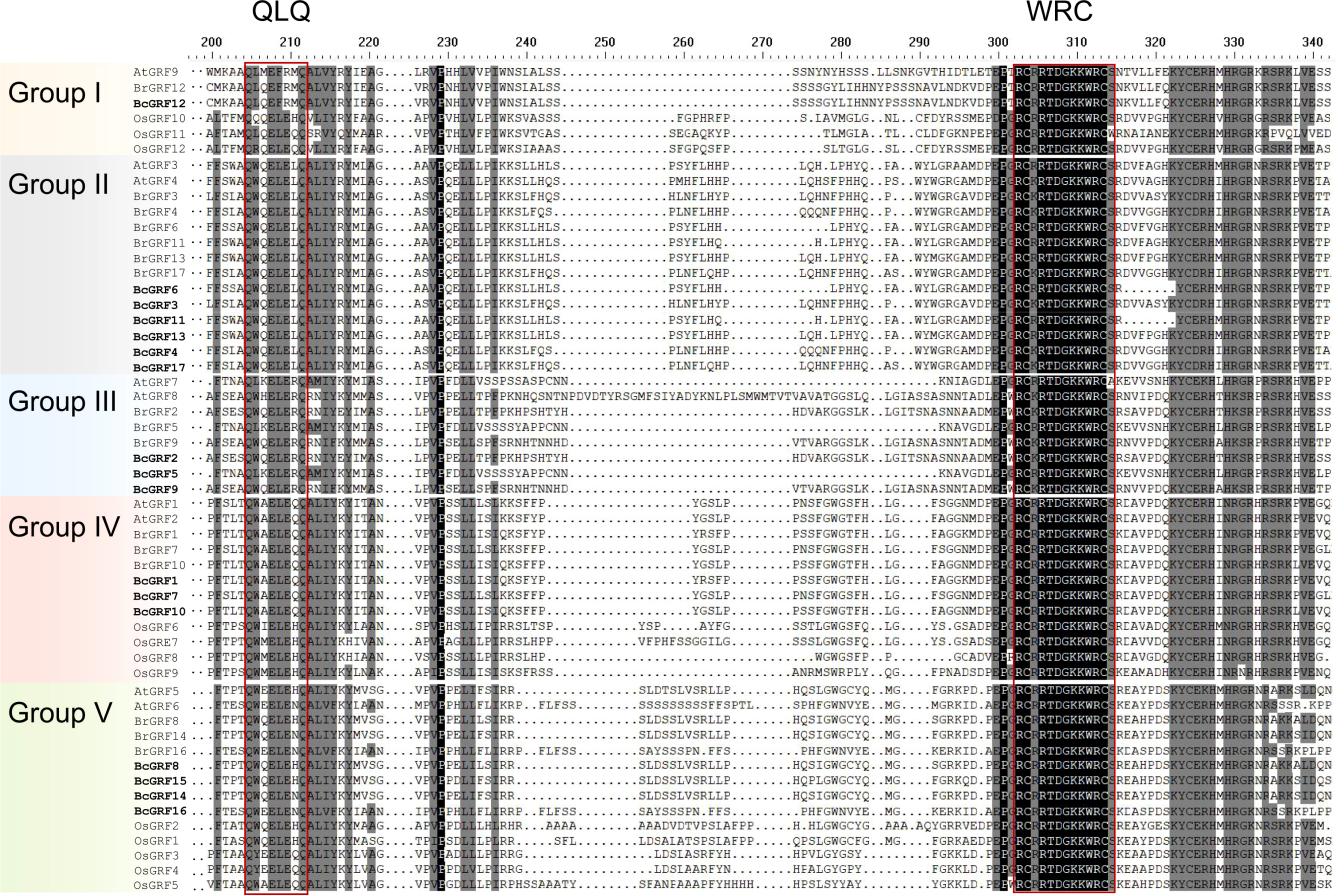
Multiple aa sequence alignment of GRF proteins from various species. The red box indicates the conserved QLQ and WRC domains. The different subfamilies are distinguished by different colors.

To infer the evolutionary relationships within the BcGRF family, 53 GRF protein sequences from *A. thaliana*, *O. sativa*, *B. campestris*, and *B. rapa* were used to construct a phylogenetic tree (**Figure 2**). The 53 GRFs were divided into five subfamilies (I–V) as follows: I included one AtGRF, one BrGRF, one BcGRF, and three OsGRFs, II included two AtGRFs, six BrGRFs, and six BcGRFs, III included two AtGRFs, three BrGRFs, and three BcGRFs, IV included two AtGRFs, three BrGRF, three BcGRFs, and four OsGRFs, and V included two AtGRFs, three BrGRFs, four BcGRFs, and five OsGRFs. A multiple aa sequence alignment of the 53 GRF proteins revealed that the sequences of *B. campestris* were highly similar to those of other species (**Figure 3**). The GRF proteins contained two highly conserved sequences at their N- and C-termini. Sequences in subfamilies II and IV were more conserved than those in other subfamilies, as evidenced by the low number of aa mutations. Interestingly, AtGRF9, BcGRF12, and BrGRF12 all had substitutions in their QLQ domains, where Leu in the middle position was replaced by Phe. Finally, a zinc finger motif (CCCH) was detected near the C-terminal WRC domain in all GRF proteins.

### 3.3 Analysis of gene structure and conserved motifs in *BcGRF* genes

**Figure 4:**
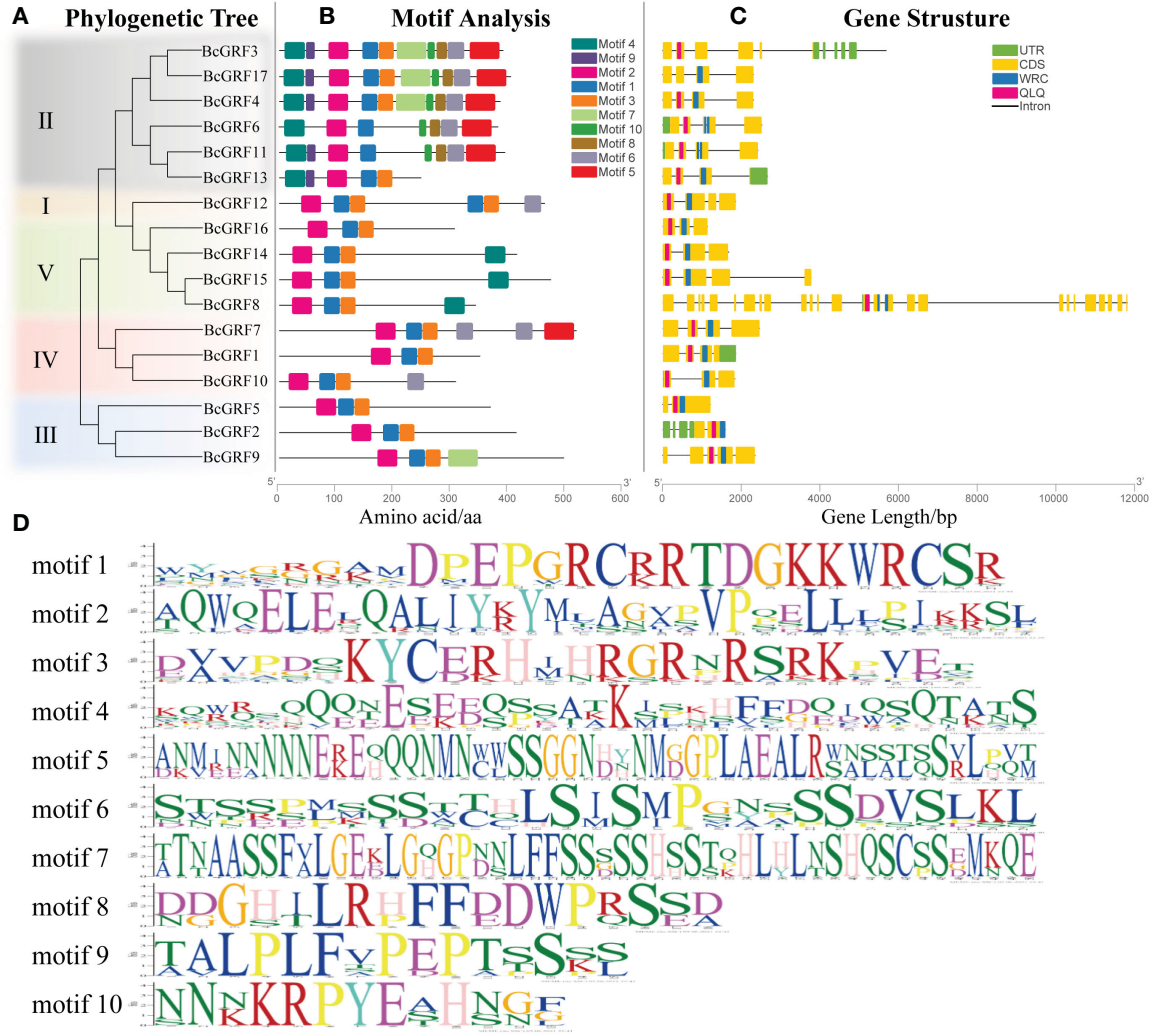
The phylogenetic relationships, gene structures, and architectures of conserved protein motifs in the *BcGRF* genes of *B*. *campestris*. **(A)** The phylogenetic tree was constructed based on full-length aa sequences of 17 BcGRF proteins using MEGA 7. The different subfamilies are distinguished by colors. **(B)** The motif composition of BcGRF proteins. The motifs (1–10) are displayed in boxes with different colors. The protein lengths can be estimated using the scale at the bottom. **(C)** The exon–intron structures of the *BcGRF* genes. The green boxes indicate untranslated 5′-UTR and 3′-UTR regions, the yellow boxes indicate exons, and the gray lines indicate introns. The conserved *GRF* domains are highlighted in red and blue boxes. **(D)** The sequence information for each motif.

Structural analysis of the *BcGRF* genes was conducted using the GSDS to visualize the CDSs, untranslated regions (UTRs), introns, and conserved domains (QLQ and WRC), according to their phylogenetic relationships (**Figure 4A**). *BcGRF* family members with close evolutionary relationships shared similar gene structures (**Figure 4C**). All *BcGRF* genes contained introns, with intron numbers ranging from one to a dozen (seven genes contained three introns, four genes contained four introns, three genes contained two introns, two genes contained one intron, and one gene contained five introns). *BcGRF8* had the longest gene sequence and contained a dozen introns. Mapping of the unique conserved domains showed that *BcGRF* contained one each of the QLQ and WRC domains. The MEME tool was used to predict and analyze the motif composition of the 17 BcGRF proteins (**Figure 4B**). All BcGRF proteins contained three motifs (motifs 1, 2, and 3), with motif 2 corresponding to the QLQ conserved domain and motif 1 corresponding to the WRC conserved domain (**Figure 4D**). BcGRFs in subfamily II had the largest number of motifs, with three of the proteins containing 10 motifs. The N-terminus of subfamily II proteins contained motif 4 (**Figure 4A, B** **)**. The C-terminus of subfamily II proteins contained different numbers of short aa domains of GGPL (motif 8), TQL (motif 6), and FFD (motif 5), suggesting that members of subfamily II may have specific functions distinct from those of other BcGRFs. Some BcGRFs showed unique subfamily patterns. For example, the C-terminus of BcGRF10, BcGRF7, and BcGRF9 each contained a specific motif, motifs 6, 5, and 7, respectively. Notably, BcGRF12 contained two conserved WRC domains at its C-terminus (**Figure 4B**). Finally, BcGRFs within the same subfamily had the same conserved motifs, validating the results of the phylogenetic analysis and illustrating the reliability of subfamily classification. The gene structures differed among subfamilies, which implies that BcGRFs have different functions.

### 3.4 Synteny analysis of *BcGRF* genes

To further understand the evolution and differentiation of *GRF* family genes, we analyzed the chromosomal distribution, synteny, and evolution of the 17 *BcGRF* genes (**Figure 1A**). We identified 16 homologous gene pairs resulting from gene duplications. The sequences of duplicated gene pairs were highly similar, suggesting that they are involved in the regulation of analogous biological processes. A synteny analysis of *GRF* gene pairs between the *B. campestris* and *A. thaliana* chromosomes revealed 17 homologous gene pairs between the two species (**Figure 1B**). Moreover, most of these *GRF* gene pairs had a cross-collinear relationship. Each *AtGRF* gene had a syntenic relationship with between one to three *BcGRF* genes, spanning less than three chromosomes. The synteny analysis showed that the expansion of the *GRF* gene family in flowering Chinese cabbage was mainly driven by gene duplications.

### 3.5 Correlation between expression patterns of *BcGRF* genes and N metabolism-related genes

**Figure 5:**
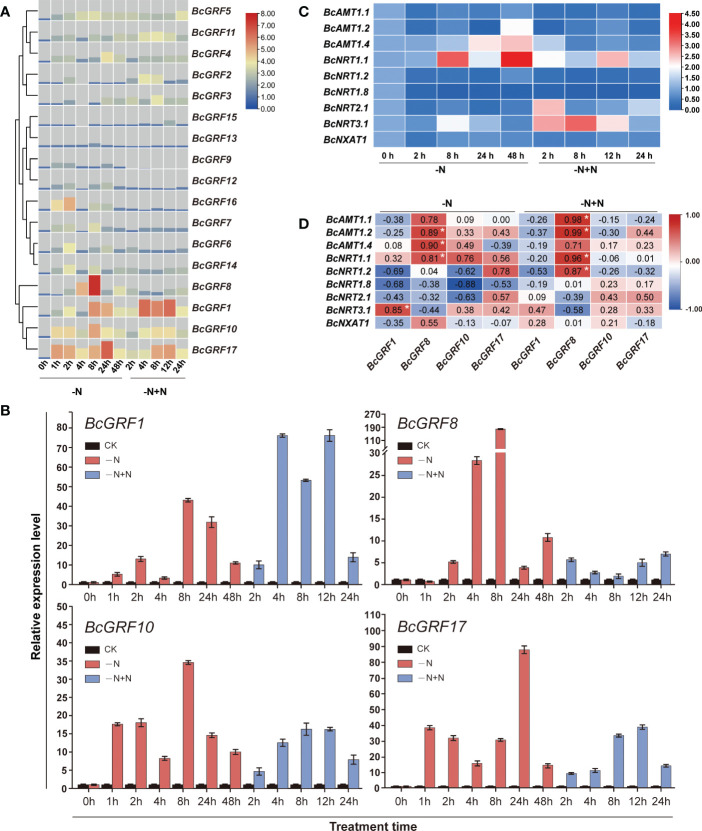
**(A)** The RT-qPCR-based expression profiles of *BcGRF* genes following N treatment. The expression of *BcGRF* genes at the start of the treatment was set to 1.0, and *GAPDH* was used as an internal reference gene. -N: N deficiency; -N+N: restoration of N supply after 48 h of N deficiency. The 2^–ΔΔCt^ method was used to evaluate the relative expression, and Log2 values were used to generate the heatmap. **(B)** The expression levels of *BcGRF1*, *BcGRF8*, *BcGRF10*, and *BcGRF17* under the different N treatments. The error bars represent standard errors. -N represents N deficiency and -N+N represents the restoration of N supply after 48 h of N deficiency. The 2^–ΔΔCt^ method was used to evaluate the relative expression. These are the same data as used in panel **(A, C)** Heatmaps showing the relative expression levels of N-related genes following N treatments. -N represents N deficiency and -N+N represents the restoration of N supply after 48 h of N deficiency. The 2^–ΔΔCt^ method was used to evaluate the relative expression, and Log2 values were used to generate the heatmap. **(D)** The correlations between the expression levels of the four key *GRF* genes and N metabolism-related genes using Pearson correlation tests. The numbers in the figure are correlation coefficients. -N represents N deficiency and -N+N represents the restoration of N supply after 48 h of N deficiency. Red indicates a positive correlation, blue indicates a negative correlation, and white indicates no correlation. The white asterisks indicate significance (*P < 0.05, Student’s *t*-test).

We analyzed the sensitivity of *BcGRF* genes to N deficiency and re-supply. In general, the *BcGRF* gene increased upon exposure to N deficiency, followed by a return to basal levels. *BcGRF1*, *8*, *10*, and *17* expression increased the most in response to N deficiency, particularly at 8 h after treatment (**Figure 5A**). Among these four genes, *BcGRF8* was the most significantly upregulated by N deficiency (178.6-fold increase at 8 h *vs*. 0 h) (**Figure 5B**). The N supply was restored after 48 h of N deficiency. After N re-supply for 12 h, the expression of these four *BcGRF* genes tended to decrease, except for *BcGRF8* (**Figure 5B**). However, their expression levels remained higher than those before the N deficiency treatment (0 h).

To explore whether the expression patterns of the above four *BcGRF* genes were related to expression patterns of N metabolism-related genes, we evaluated the levels of three ammonia transporter genes (*BcAMT1.1*, *BcAMT1.2*, and *BcAMT1.4*), five ${\text{NO}}_{3}^{-}$ transporter genes (*BcNRT1.1*, *BcNRT1.2*, *BcNRT1.8*, *BcNRT2.1*, and *BcNRT3.1*), and a ${\text{NO}}_{3}^{-}$ efflux transporter gene (*BcNXAT1*) in response to N treatment (**Figure 5C**). These genes are responsible for the absorption and transport of ${\text{NH}}_{4}^{+}$ and ${\text{NO}}_{3}^{-}$ and are key genes in the N metabolic pathway (Zhu et al., 2021). *BcAMT1.2*, *1.4*, and *BcNRT1.1*, *2.1*, and *3.1* levels were upregulated under N deficiency. In particular, *BcNRT1.1* expression was the highest after 48 h of N deficiency treatment. Next, we analyzed the correlations between the expression levels of the four *BcGRF* genes and N metabolism-related genes under N deficiency and re-supply. We found that *BcGRF8* levels were significantly positively correlated with *BcAMT1.2, BcAMT1.4*, and *BcNRT1.1* levels under N deficiency. After N re-supply, *BcGRF8* expression showed strong positive correlations with the levels of *BcAMT1.1*, *BcAMT1.2*, *BcNRT1.1*, and *BcNRT1.2* (**Figure 5D**). Overall, *BcGRF8* expression was the most sensitive to N deficiency and was significantly correlated with the expression patterns of most N metabolism-related genes.

### 3.6 BcGRF8 interacts with the *BcNRT1.1* promoter

**Figure 6:**
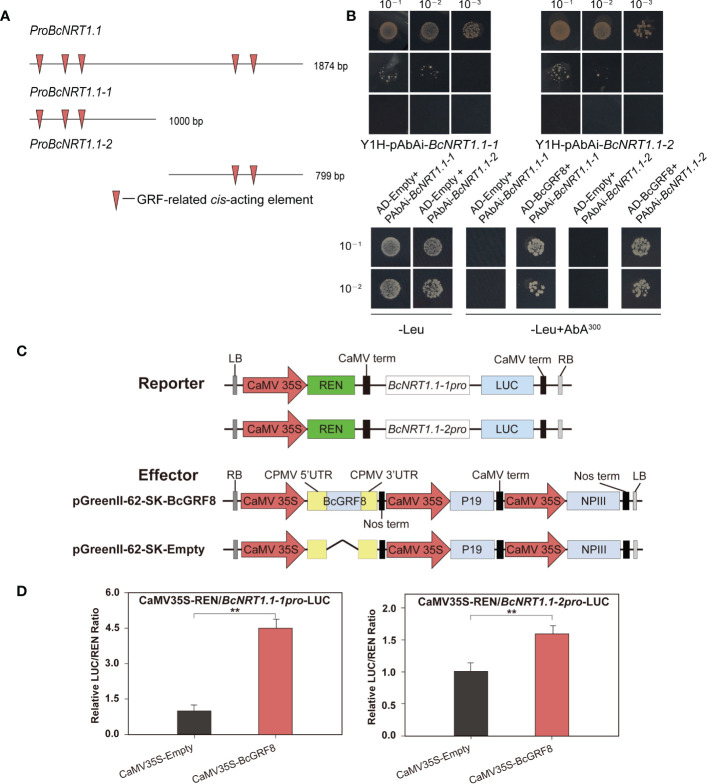
BcGRF8 regulates *BcNRT1.1* promoter activity. **(A)** GRF-related *cis*-acting elements in the *BcNRT1.1* promoter regions. **(B)** Yeast one-hybrid assay. The auto-activation of promoters was tested on a synthetically defined medium lacking Ura in the presence of AbA. The interactions were determined on a synthetically defined medium lacking Leu in the presence of AbA (-Leu+AbA^300^). Negative control: AD-Empty+*BcNRT1.1-1* promoter, AD-Empty+*BcNRT1.1-2* promoter. **(C)** Reporter and effector gene constructs. **(D)** BcGRF8 activates two *BcNRT1.1* promoter fragments. The experiments were repeated three times, and representative results from one experiment are shown. The error bars represent standard errors. (**P < 0.01, Student’s *t*-test).

To verify whether BcGRF8 has a direct targeting relationship with *BcNRT1.1*, we performed yeast one-hybrid and dual-LUC assays (**Figure 6**). We first cloned the 1874-bp *BcNRT1.1* promoter and divided it into two parts (*proBcNRT1.1-1* and *proBcNRT1.1-2*). A prediction analysis showed that *proBcNRT1.1-1* and *proBcNRT1.1-2* contained three and two GRF-related *cis*-acting elements, respectively (**Figure 6A**). The yeast one-hybrid assay showed that BcGRF8 was stably bound to the *BcNRT1.1* promoter, driving the transcription of the Leu-expressing locus in the yeast system **(** **Figure 6B** **)**. For the dual-LUC assay, two reporter plasmids (pGreenII 0800-*proBcNRT1.1-1-*LUC and pGreenII 0800-*proBcNRT1.1-2-*LUC) and effector plasmids (pGreenII 62-SK-BcGRF8 and pGreenII 62-SK-Empty) were constructed (**Figure 6C**). BcGRF8 strongly enhanced the *BcNRT1.1* promoter activity by interacting with the two *BcNRT1.1* promoter regions in tobacco leaves (**Figure 6D**). These findings preliminarily suggested that BcGRF8 is a key TF in the regulation of the ${\text{NO}}_{3}^{-}$ transport and metabolic processes in flowering Chinese cabbage.

### 3.7 Spatiotemporal expression pattern and subcellular localization of *BcGRF8*

**Figure 7:**
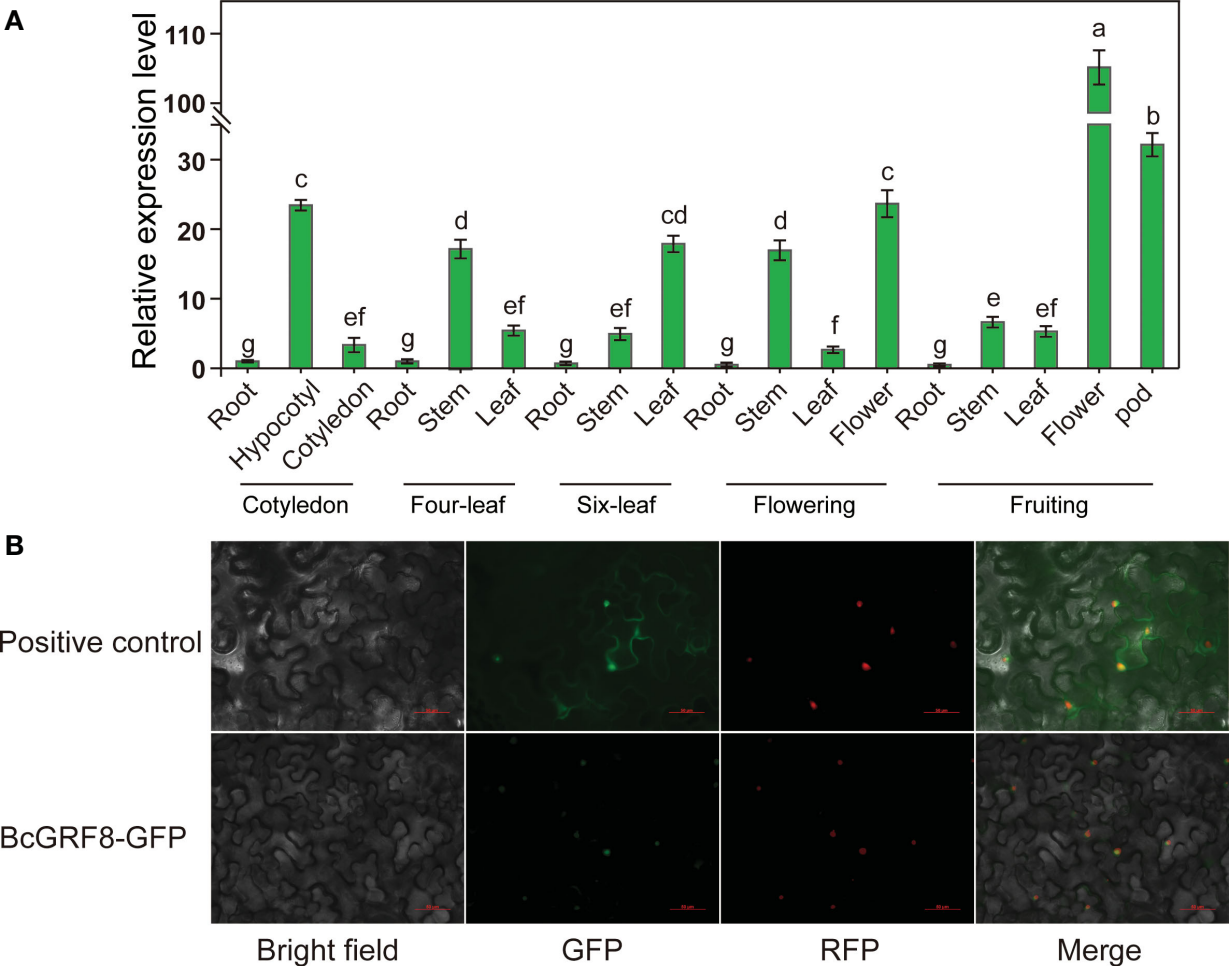
**(A)** The RT-qPCR-based expression profiles of *BcGRF8* in various tissues. The expression level of *BcGRF8* in roots at the cotyledon stage was set to 1.0 and *BcGAPDH* was used as an internal reference gene. The 2^–ΔΔCt^ method was used for data analysis, and log2 values were used to generate the histogram. Error bars represent standard errors. Different lowercase letters indicate significant differences at the *P* < 0.05 level. **(B)** Subcellular localization of BcGRF8. Bar = 50 μm.

To further characterize the role of *BcGRF8* in flowering Chinese cabbage, its spatiotemporal expression pattern was analyzed in different tissues over five developmental stages. The *BcGRF8* gene was expressed in all organs of flowering Chinese cabbage; however, expression levels differed significantly among organs (**Figure 7A**). *BcGRF8* was highly expressed in the hypocotyls and stems throughout the growing season, except for the fruiting stage. During the fruiting stage, *BcGRF8* expression was the highest in the flowers, and *BcGRF8* expression was also the highest at this stage in the entire growth period. However, *BcGRF8* expression was the lowest in the roots throughout the growth period. A subcellular localization analysis showed that the *BcGRF8* was localized in the nucleus (**Figure 7B**).

### 3.8 BcGRF8 promotes plant growth under both ${\text{NO}}_{3}^{-}$ -rich and -poor conditions

**Figure 8:**
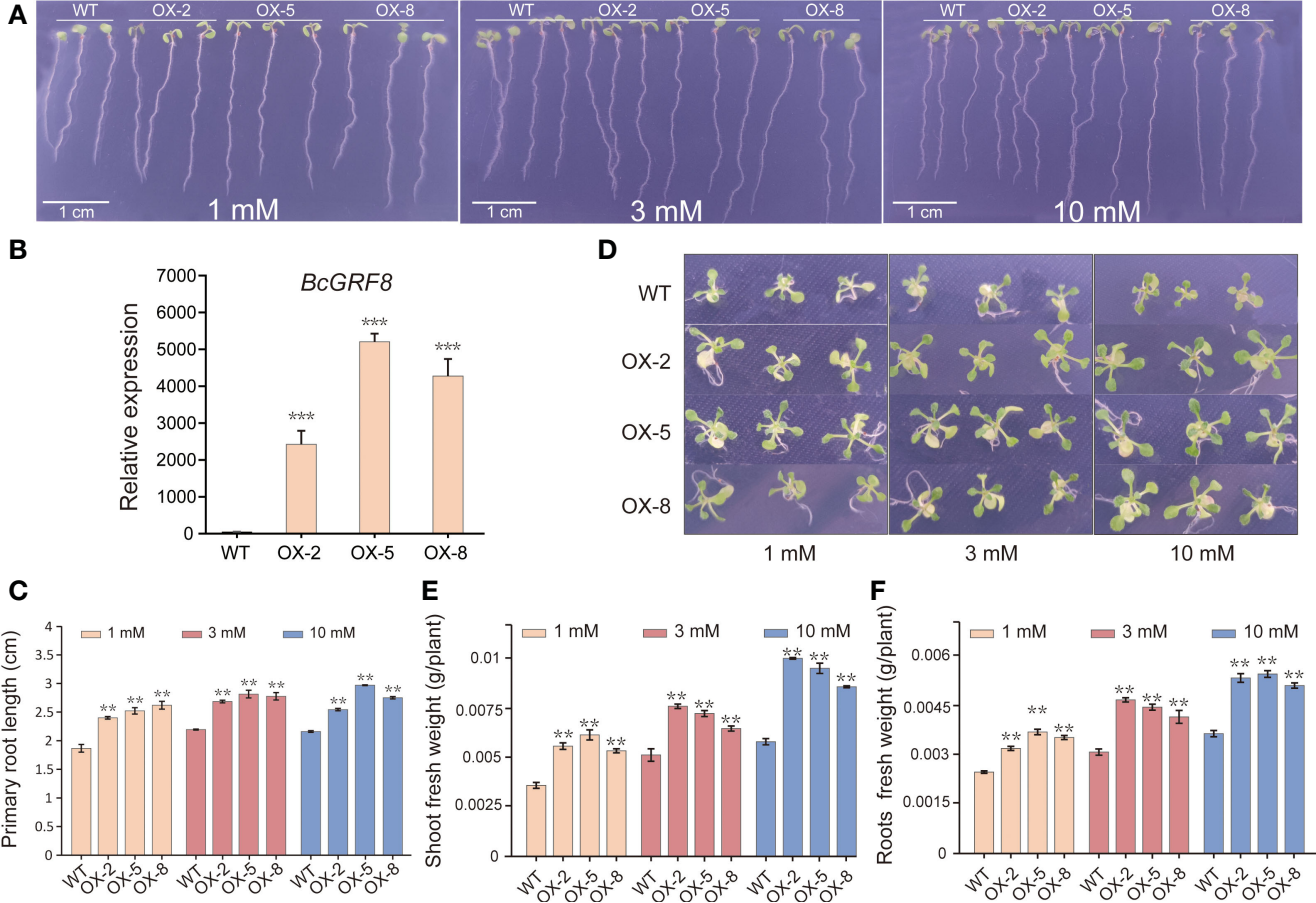
BcGRF8 improves plant growth under both ${\text{NO}}_{3}^{-}$ -rich and -poor conditions. **(A)** The phenotypes of 7-day-old WT and *BcGRF8*-overexpressing seedlings grown vertically on plates with medium containing different concentrations of ${\text{NO}}_{3}^{-}$ . Bar = 1 cm. **(B)** The expression levels of BcGRF8 in WT and *BcGRF8*-overexpressing Arabidopsis seedlings. The values are means ± SDs of three replications. (***P < 0.001 vs. WT). **(C)** The primary root lengths of 7-day-old plants grown under different ${\text{NO}}_{3}^{-}$ conditions. The error bars indicate standard deviations (SDs) of biological triplicates (**P < 0.01). **(D)** The phenotypes of 20-day-old WT and *BcGRF8*-overexpressing seedlings grown on plates with medium containing different concentrations of ${\text{NO}}_{3}^{-}$ . **(E, F)** Shoot **(E)** and root **(F)** FWs of 20-day-old seedlings grown under different ${\text{NO}}_{3}^{-}$ conditions. The values are means ± SDs of three replications. (**P < 0.01 *vs*. WT).

To investigate the effects of BcGRF8 expression on plant growth, we generated 35S:BcGRF8 cDNA transgenic Arabidopsis plants. Three T3 transgenic Arabidopsis lines with the highest *BcGRF8* expression levels (OX-2, OX-5, and OX-8) were selected for the experiments (**Figure 8B**). WT and T3 transgenic Arabidopsis were grown on a modified MS solid medium with different concentrations of ${\text{KNO}}_{3}^{-}$ as the sole inorganic N source. We measured the primary root lengths of 7-day-old seedlings using a vertical growth assay. OX-2, OX-5, and OX-8 plants exhibited rapid root growth on both ${\text{NO}}_{3}^{-}$ -rich and -poor media. The roots of OX-2, OX-5, and OX-8 plants were significantly longer than those of WT plants under 1–10 mM ${\text{NO}}_{3}^{-}$ (**Figures 8A, C**). In addition, the FW of 20-day-old seedlings on a modified MS solid medium was significantly higher for OX-BcGRF8 plants than for WT seedlings and increased with increasing ${\text{NO}}_{3}^{-}$ concentration (**Figures 8D-F**). These results indicated that *BcGRF8* significantly promotes the growth of Arabidopsis shoots and roots under both ${\text{NO}}_{3}^{-}$ -rich and -poor conditions.

### 3.9 BcGRF8 alters the number of lateral roots and reduces the ${\text{NO}}_{3}^{-}$ N content in Arabidopsis

**Figure 9:**
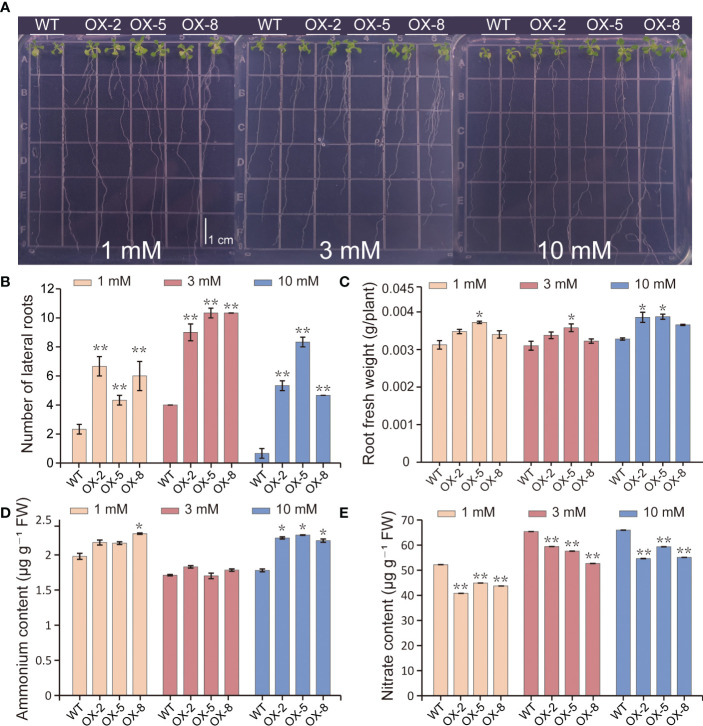
BcGRF8 promotes lateral root development and reduces the ${\text{NO}}_{3}^{-}$ content in Arabidopsis. **(A)** The phenotypes of 20-day-old seedlings grown vertically on plates containing different concentrations of ${\text{NO}}_{3}^{-}$ . **(B–E)** The number of lateral roots **(B)**, root FW **(C)**, ${\text{NH}}_{4}^{+}$ content **(D)**, and ${\text{NO}}_{3}^{-}$ content **(E)** of Arabidopsis under different ${\text{NO}}_{3}^{-}$ conditions. The values are means ± SDs of three replications. (*P < 0.05, **P < 0.01 vs. WT).

To verify whether the overexpression of *BcGRF* influences the root architecture under different ${\text{NO}}_{3}^{-}$ treatments, we evaluated the roots of WT and OX-2, OX-5, and OX-8 seedlings grown vertically on agar media containing different concentrations of ${\text{NO}}_{3}^{-}$ . *BcGRF8* overexpression led to increased root FWs compared with those of WT plants under both ${\text{NO}}_{3}^{-}$ -rich and -limited conditions (**Figures 9A, C**). Since *NRT1.1*, the target gene of BcGRF8, is involved in lateral root development and is regulated by exogenous N, we observed lateral root development of Arabidopsis seedlings under different ${\text{NO}}_{3}^{-}$ concentrations. The number of lateral roots in *BcGRF8*-overexpressing plants was higher than that in WT plants under both ${\text{NO}}_{3}^{-}$ -rich and -limiting conditions (**Figure 9B**). To determine the effect of *BcGRF8* overexpression on N metabolism, we detected the ${\text{NH}}_{4}^{+}$ and ${\text{NO}}_{3}^{-}$ contents in *BcGRF8*-overexpressing and WT Arabidopsis. The ${\text{NH}}_{4}^{+}$ N contents of OX-2, OX-5, and OX-8 seedlings were significantly higher than those of WT seedlings under 10 mM ${\text{NO}}_{3}^{-}$ ; however, no significant difference was observed under the other two N treatments (**Figure 9D**). The ${\text{NO}}_{3}^{-}$ contents of OX-2, OX-5, and OX-8 seedlings were significantly lower than those of WT plants under both N-rich and -limiting conditions (**Figure 9E**). These results indicated that the increase in *BcGRF8* transcript abundance led to an increase in the number of lateral roots and a significant reduction in the ${\text{NO}}_{3}^{-}$ content in Arabidopsis.

### 3.10 BcGRF8 broadly regulates genes related to N uptake, utilization, and signaling

**Figure 10:**
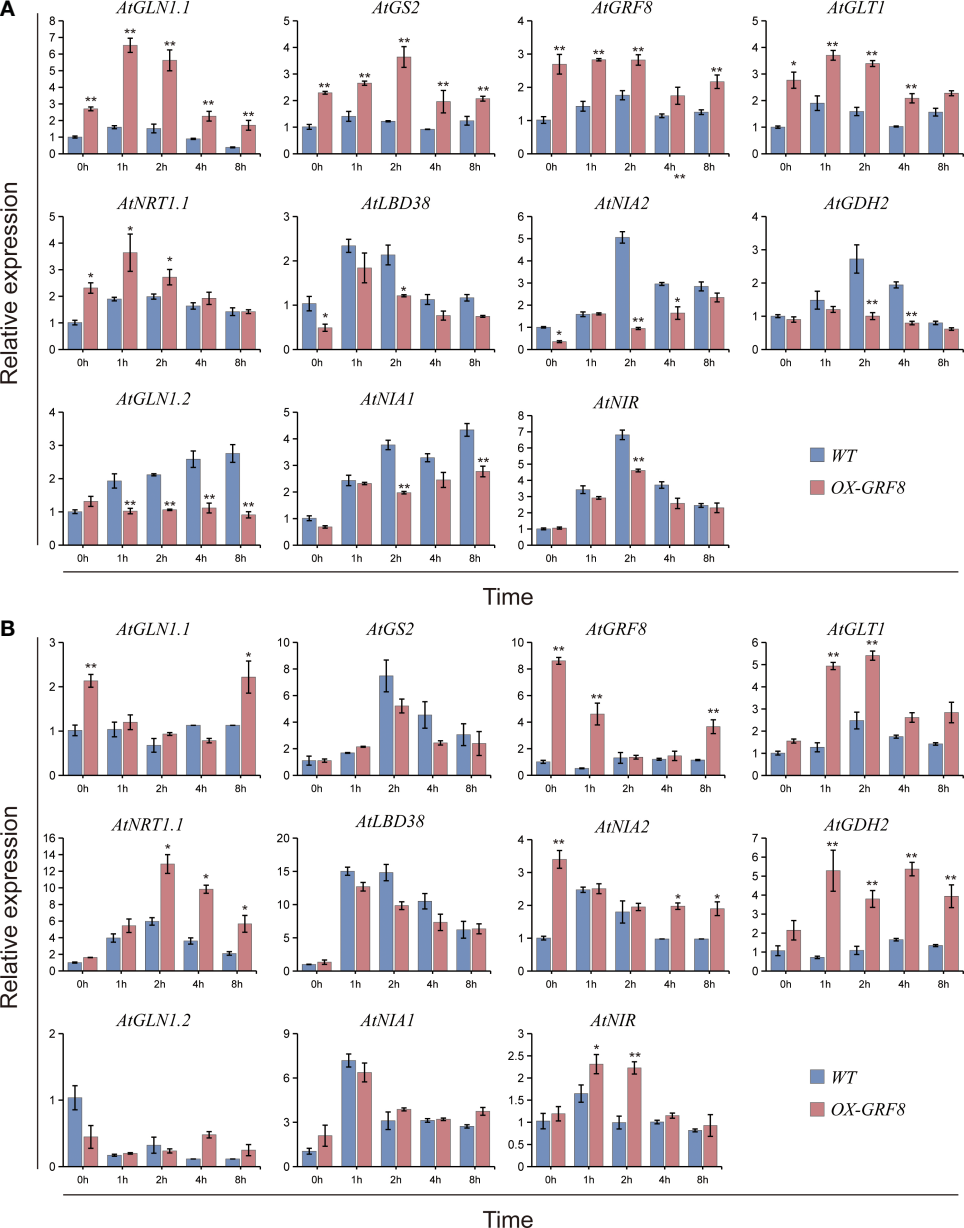
BcGRF8 broadly regulates genes related to N uptake, utilization, and signaling in Arabidopsis shoots **(A)** and roots **(B)**. Briefly, 20-day-old seedlings in normal hydroponic conditions were transferred to N-free nutrient solution for 3 days and harvested for qRT-PCR after they were re-supplied with 3 mM ${\text{NO}}_{3}^{-}$ for 0, 1, 2, 4, or 8 h *AtACTIN2* (At3g18780) was used as an internal control. AtGLN1.1, glutamine synthetase 1.1; GS2, glutamine synthetase 2; AtGLT1, glutamate synthetase 1; AtLBD38, lateral organ boundary domain 38; AtNIA2, ${\text{NO}}_{3}^{-}$ reductase 2; AtGDH2, glutamate dehydrogenase; AtGLN1.2, glutamine synthetase 1.2; AtNIA1, ${\text{NO}}_{3}^{-}$ reductase 1; AtNIR, ${\text{NO}}_{2}^{-}$ reductase. The values are means ± SDs of three replications. (*P < 0.05, **P < 0.01 *vs*. WT).

To further assess the role of BcGRF8 in N uptake and assimilation in plants, we performed RT-qPCR analysis to investigate the transcript levels of genes related to ${\text{NO}}_{3}^{-}$ assimilation and signaling, including *AtGLN1.1*, *AtGS2*, *AtGLT1*, *AtNRT1.1*, *AtLBD38*, *AtNIA2*, *AtGDH2*, *AtGLN1.2*, *AtNIA1*, and *AtNIR*, in seedlings resupplied with 3 mM ${\text{NO}}_{3}^{-}$ for 0, 1, 2, 4, or 8 h. After N starvation for 3 days, the transcript levels of *AtGLN1.1*, *AtGS2*, *AtGRF8*, *AtGLT1*, and *AtNRT1.1* in shoots of *BcGRF8*-overexpressing seedlings were significantly higher than those in WT shoots; the expression of these genes initially increased and then decreased in *BcGRF8*-overexpressing seedlings and was significantly higher than that in WT plants after re-supply of 3 mM ${\text{NO}}_{3}^{-}$ (**Figure 10A**). In contrast, *AtLBD38*, *AtNIA2*, *AtGDH2*, *AtGLN1.2*, and *AtNIA1* showed the opposite trend; their expression levels were significantly lower in *BcGRF8*-overexpressing seedlings than in WT seedlings (**Figure 10A**). In Arabidopsis roots, the expression levels of 11 N-related genes changed after ${\text{NO}}_{3}^{-}$ was re-supplied in both *BcGRF8*-overexpressing and WT seedlings (**Figure 10B**). *AtGLN1.1*, *AtGRF8*, *AtGLT1*, *AtNRT1.1*, *AtNIA2*, *AtGDH2*, and *AtNIR* expression levels in *BcGRF8*-overexpressing seedlings were significantly higher than those in WT seedlings after re-supply of 3 mM ${\text{NO}}_{3}^{-}$ , whereas the expression levels of other genes did not significantly differ between WT and *BcGRF8*-overexpressing seedlings (**Figure 10B**). These results show that increased expression of *BcGRF8* induced widespread changes in the expression of N-related genes in Arabidopsis shoots and roots, suggesting that BcGRF8 is a critical TF for ${\text{NO}}_{3}^{-}$ assimilation and signaling.

## 4 Discussion

N is one of the most important macronutrients that are involved in plant growth and development as well as metabolic processes. N is the key constituent of proteins, nucleotides, chlorophyll, metabolites, and cellular components, hence its availability in the soil directly influences crop growth and production. The economic importance of N as one of the most important inputs in crop production was established over 100 years ago (Sutton et al., 2011). Therefore, it is necessary to expand our basic knowledge of the molecular regulatory networks related to plant N metabolism, apply the concept of integrated nutrient management to optimize N fertilizer input, and fully exploit the innate biological potential of crops (Jiao et al., 2018; Gong et al., 2020) to ultimately achieve agricultural sustainability and intensification by improving NUE and reducing environmental costs.

GRFs regulate plant growth and development and the response to hormonal and stress cues (Wu et al., 2022). Evidence suggests that GRFs are key TFs in the regulation of N metabolism in some plant species (Ueda and Yanagisawa, 2018). In the present study, 17 *BcGRF* genes were identified based on whole-genome sequencing data analysis of flowering Chinese cabbage. The *GRF* gene family in flowering Chinese cabbage is more diverse than that in *A. thaliana* (9 genes) (Kim et al., 2003), *Camellia japonica* (6 genes) (Wu et al., 2017), *C. sativus* (8 genes) (Zhou et al., 2018), *Brachypodium sylvaticum* (10 genes) (Filiz et al., 2014), *O. sativa* (12 genes) (Choi et al., 2004), *S. lycopersicum* (13 genes) (Khatun et al., 2017), and *Z. mays* (14 genes) (Wu et al., 2014), but less diverse than that in *B. napus* (35 genes) (Ma et al., 2017), *T. aestivum* (30 genes) (Huang et al., 2021), *A. hypogaea* (24 genes) (Zhao et al., 2019), and *Nicotiana tabacum* (25 genes) (Zhang et al., 2018). This variation indicates that the *GRF* family underwent extensive expansion and diversification in these plant species. As expected, the number of *BcGRF* genes in flowering Chinese cabbage was the same as that in Chinese cabbage (i.e., 17) (Wang et al., 2014). This is presumably because flowering Chinese cabbage is a subspecies of Chinese cabbage. Gene mapping showed that the *BcGRF* genes are distributed on seven chromosomes, whereas the *BrGRF* genes are distributed on eight chromosomes (Wang et al., 2014). Moreover, the specific positions of *GRF* genes on individual chromosomes differ, which may be explained by divergence among subspecies and the domestication history of *B. rapa*.

The *BcGRF* gene family members were classified into five subfamilies based on a phylogenetic analysis including sequences from *A. thaliana, B. rapa*, and *O. sativa*. The majority of *AtGRF*, *BrGRF*, and *BcGRF* genes belonged to the same subfamilies, whereas no *OsGRF* genes were found in groups II and III. *GRF* genes in *B. campestris* and *A. thaliana* were closely related. BcGRF proteins from multiple species contained the QLQ and WRC domains. A previous study of 410 *GRFs* from 45 species showed that 22 *GRFs* contained two WRC conserved domains (Leila et al., 2020). The same study revealed that *BcGRF12* contains one QLQ domain and two WRC domains, consistent with the structural features of *AtGRF9* (Kim et al., 2003) and *BrGRF12* (Wang et al., 2014). Moreover, we detected a Leu → Phe substitution in the QLQ domains of *BcGRF12* and *AtGRF9*; this substitution has been also detected in tomato (Khatun et al., 2017) and the tea plant (Wu et al., 2017). The substitution may result in distinct functions from those of other GRF proteins; however, this remains to be confirmed. Moreover, the C-termini of BcGRF proteins in subfamily II (except for BcGRF13) contained the TQL, FFD, and GGPL conserved domains. These domains with low conservation also exist in the GRF proteins of Chinese cabbage, cucumber, and tomato and are mostly located in the C-terminus (Liu et al., 2012). Hence, functional diversity among BcGRF proteins can be predicted based on the diversity of C-terminal domains.

Generally, gene families expand through gene and gene fragment duplications (Chang and Duda, 2012). *Brassica* species (e.g., *B. rapa* and *B. juncea*) have more genes than *A. thaliana* due to a genome-wide tripling event in *Brassica* after its divergence (Liu et al., 2013; Wang et al., 2014). However, the number of *GRF* genes in *B. campestris* was not three times that in Arabidopsis, indicating that several gene loss events have occurred in this lineage. The Arabidopsis GRF-interacting factor 1 (*gif1*) mutant has a longer stem than WT Arabidopsis (Ercoli et al., 2018). Overexpression of *AtGRF3* promoted leaf growth, whereas *AtGRF9* overexpression suppressed leaf growth by inhibiting the proliferation of leaf primordia (Kim et al., 2003). The QLQ domain of GRF proteins interacts with the SNH domain of GIF proteins to form a complex that activates the expression of downstream target genes (Ercoli et al., 2018). The orthologs of *AtGRF1*, *AtGRF3*, and *AtGRF9* in flowering Chinese cabbage, i.e., *BcGRF1*, *6*, *7*, *12*, and *13*, may contribute to plant cell proliferation to regulate the growth and development of stems and leaves.

In rice, N utilization can be improved by regulating carbon and N metabolism *via* a complex regulatory network (Li et al., 2018b; Zhang et al., 2020). OsGRF4 interacts with OsGIF1 to activate the transcription of downstream N absorption-related genes (e.g., *AMT1.1* and *AMT1.2*) and N assimilation-related genes (*GS1*) in rice (Ma and Liu, 2018). In peanut plants, N deficiency significantly reduced the root surface area, root vitality, primary root length, and lateral root number, which may be related to the downregulation of *GRF* expression *via* miR396 (Li, S. et al., 2021). Therefore, we explored the effect of N deficiency and re-supply on the expression of *BcGRF* genes and found that *BcGRF1*, *8*, *10*, and *17* were sensitive to changes in N. Moreover, the expression levels of N metabolism-related genes, e.g., *BcNRT1.1*, *BcNRT1.4*, and *BcNRT3.1*, increased significantly following N treatment. Under N deficiency, *BcGRF8* expression showed significant positive correlations with that of *BcAMT1.2*, *BcAMT1.4*, and *BcNRT1.1*, whereas after N re-supply, it was strongly positively correlated with *BcAMT1.1, BcAMT1.2, BcNRT1.1*, and *BcNRT1.2* expression. Interestingly, *BcGRF8* was the most sensitive to N deficiency and was significantly correlated with the expression patterns of most of the N metabolism-related genes. Based on these findings, we hypothesize that the *BcGRF8* may act as a transcriptional activator to regulate the expression of downstream N metabolism-related genes, thereby participating in N absorption and utilization in flowering Chinese cabbage.

Despite increasing evidence that GRFs play a role in N absorption and transport, the specific regulatory pathways require further investigation. Poplars overexpressing *PpnGRF5-1* had significantly lower transcript levels of *PagLBD38* than WT plants, whereas *PagNRT1.5* and *PagNRT1.7* were upregulated in apical buds (Wu et al., 2021). AtLBD38 inhibits many known N-responsive genes in Arabidopsis, including *AtNRT* (Rubin et al., 2009). However, it was not clear whether GRF directly regulates the *NRT* gene. The current study confirmed that BcGRF8 binds to the *BcNRT1.1* promoter and promotes transcription. NRT1.1 and NRT1.2 are important members of the low-affinity transport system, which is responsible for the absorption and transport of ${\text{NO}}_{3}^{-}$ in plants (Leran et al., 2015). NRT1.1 also functions as a high-affinity transporter and is involved in root development (taproot, lateral root, and root hair density). Increased expression of *NRT1.1* increases the root absorption area, improves the nutrient absorption capacity, and alleviates the inhibitory effect of low-nutrient stress on plant growth (Bouguyon et al., 2012; Bouguyon et al., 2016; Canales et al., 2017). Therefore, modification to the upstream regulatory factor BcGRF8 to regulate *NRT* gene expression may be a strategy to improve NUE in plants.

To further study the biological function of BcGRF8, we generated *BcGRF8*-overexpressing Arabidopsis lines. *BcGRF8* overexpression increased the primary root length and shoot and root FW in Arabidopsis under both ${\text{NO}}_{3}^{-}$ -rich and -poor conditions. Well-developed lateral roots are beneficial for nutrient absorption and alleviate the inhibitory effect of low-nutrient stress on plant growth (Chang et al., 2022). Arabidopsis AtNRT1.1 is involved in lateral root development and is regulated by the concentration of exogenous N (Beeckman and Friml, 2010). Local ${\text{NO}}_{3}^{-}$ availability significantly promoted the growth of lateral roots on the N supply side in Arabidopsis (Remans et al., 2006). We found that *BcNRT1.1* is a target gene of BcGRF8. Hence, we speculated that the upregulation of GRF8 may indirectly regulate lateral root development by modulating the transcript abundance of *NRT1.1* in plants. We found that *BcGRF8* overexpression indeed significantly increased the number of lateral roots in Arabidopsis under different concentrations of ${\text{NO}}_{3}^{-}$ . *BcGRF8* overexpression increased the biomass of Arabidopsis plants in a culture medium with ${\text{NO}}_{3}^{-}$ as the sole N source, indicating that BcGRF8 may enhance the absorption or the use efficiency of ${\text{NO}}_{3}^{-}$ . *BcGRF8*-overexpressing plants had higher ${\text{NH}}_{4}^{+}$ N contents than WT plants under ${\text{NO}}_{3}^{-}$ -rich conditions, indicating that ${\text{NH}}_{4}^{+}$ , as a product of ${\text{NO}}_{3}^{-}$ assimilation, accumulated in the plants. In contrast, ${\text{NO}}_{3}^{-}$ contents in *BcGRF8-*overexpressing plants were lower than those in WT plants, suggesting that BcGRF8 enhanced the reduction reaction of ${\text{NO}}_{3}^{-}$ by inducing the expression of N assimilation-related genes in these plants.

N uptake, transport, assimilation, and signal transduction all involve complex gene regulatory networks. In the present study, BcGRF8 induced the expression of genes related to ${\text{NO}}_{3}^{-}$ uptake (*AtNRT1.1*), N assimilation (*AtGLN1.1*, *AtGS2*, *AtGLT1*, *AtNIA2*, *AtGDH2*, *AtGLN1.2*, *AtNIA1*, and *AtNIR*), and N signaling (*AtGRF8* and *AtLBD38*) in Arabidopsis shoots and roots. ${\text{NO}}_{3}^{-}$ absorbed by plants is assimilated directly in the roots or transported to the shoots through the xylem for assimilation (Nunes-Nesi et al., 2010). We found that the ${\text{NO}}_{3}^{-}$ contents of *BcGRF8-*overexpressing plants were significantly lower than those in WT plants under both ${\text{NO}}_{3}^{-}$ -rich and -limiting conditions. It is possible that *BcGRF8* overexpression led to the significant upregulation of *AtNIA2* and *AtNIR* in transgenic Arabidopsis roots. These data suggest that the transcript abundance of *BcGRF8* broadly induces N uptake and assimilation-related genes, thereby increasing the biomass and reducing the ${\text{NO}}_{3}^{-}$ content in plants.

Excessive use of N leads to the accumulation of ${\text{NO}}_{3}^{-}$ in vegetables, especially leafy vegetables, which threatens human health. Our results, which clearly demonstrate that *BcGRF8* is critical in the regulation of plant development and N metabolism, emphasize the need for continued studies of this gene in flowering Chinese cabbage and provide guidance for future studies. One limitation of this study was that we did not fully unravel the underlying mechanisms; in future studies, we will focus on the specific molecular mechanism of *BcGRF8* in regulating N metabolism in flowering Chinese cabbage. In particular, we will aim to identify the function and additional target genes of BcGRF8 in N metabolism using *BcGRF8* transgenic flowering Chinese cabbage plants generated with gene editing and protein overexpression technologies. We will seek to construct a multilayered hierarchical regulatory network mediated by BcGRF8. These studies will lay the foundation for improving N fertilizer utilization and reducing the ${\text{NO}}_{3}^{-}$ content in flowering Chinese cabbage through genetic engineering.

## 5 Conclusions

Through a genome-wide analysis of the *BcGRF* family in flowering Chinese cabbage, we identified 17 *BcGRF* genes. The *BcGRF* family genes are mainly distributed on seven chromosomes, and their evolutionary expansion was driven by gene duplication. Correlation analysis suggested that several candidate genes (*BcGRF1*, *8*, *10*, and *17*) are involved in the regulation of N metabolism in flowering Chinese cabbage. Among these, BcGRF8 is highly sensitive to N deficiency and its expression was significantly correlated with the expression patterns of most N metabolism-related genes. We further found that BcGRF8 strongly enhances *BcNRT1.1* gene promoter activity. Biological functional analysis of *BcGRF8* in Arabidopsis revealed that BcGRF8 is localized in the cell nucleus. *BcGRF8* overexpression significantly increased the FW of Arabidopsis shoots and roots, primary root length, and lateral root number. In addition, *BcGRF8* overexpression reduced the ${\text{NO}}_{3}^{-}$ content in Arabidopsis. Finally, BcGRF8 was found to broadly regulate genes related to N uptake, utilization, and signaling. This foundational study provides a framework for future research, and the results could be used for reducing the ${\text{NO}}_{3}^{-}$ content and breeding N-use-efficient crops.

## Data availability statement

The original contributions presented in the study are included in the article/**Supplementary Material**. Further inquiries can be directed to the corresponding author.

## Author contributions

SZ, BH, and JZ performed the experiments. YH, SS, and RC participated in the design of the study. SZ, GL, and YH analyzed the data, and SZ wrote the manuscript. YW, CC, YH, and SS assisted in revising the manuscript. All authors contributed to the article and approved the submitted version.
