# Supplementary material for: Genome-wide identification of BcGRF genes in flowering Chinese cabbage and preliminary functional analysis of BcGRF8 in nitrogen metabolism
Source: Front Plant Sci. 2023 Mar 9;14:1144748. doi: 10.3389/fpls.2023.1144748 (PMC10034182; doi:10.3389/fpls.2023.1144748)
Supplement: Supplementary file 1 [file Image_1.pdf]

frontiers

Supplementary Material

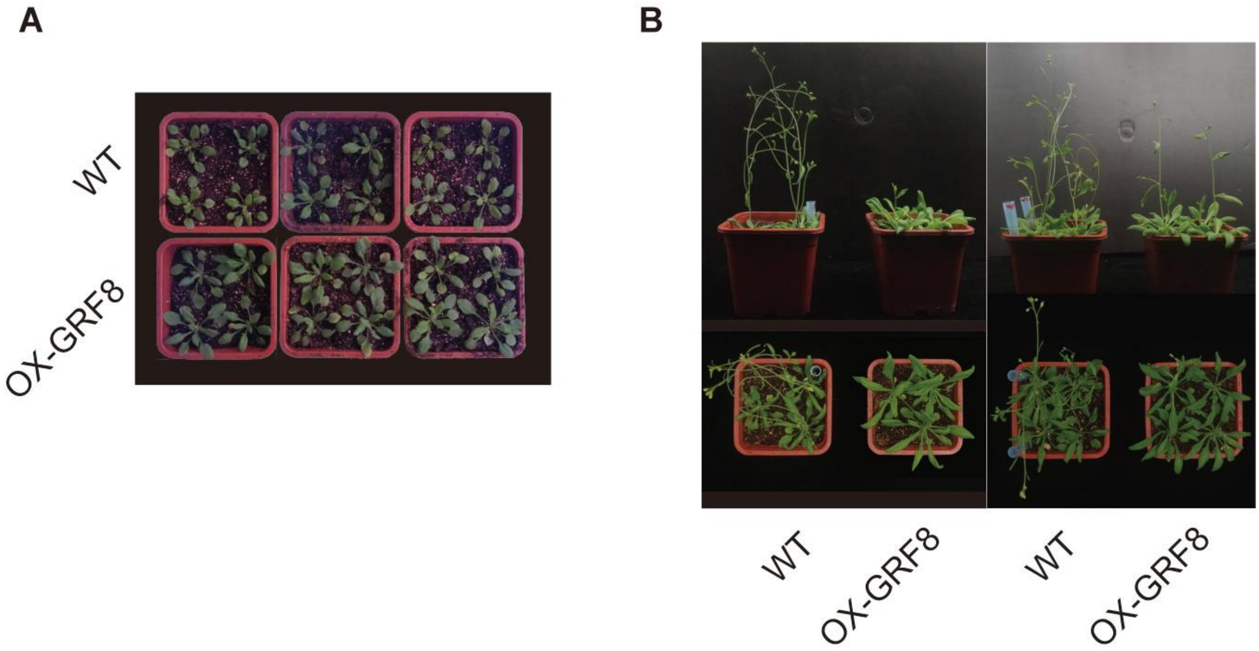

Supplementary Figure 1. Plants phenotypes of the BcGRF8-overexpression lines and WT under normal growth conditions.
